# Clonal hematopoiesis of indeterminate potential is associated with increased cardiovascular risk in chronic kidney disease

**DOI:** 10.1093/ckj/sfag141

**Published:** 2026-05-08

**Authors:** Jie Liu, Shangjian Luo, Zhiqiang Liu, Tingting Wang, Junlong Chen, Hua Gan, Dongying Zhang, Jun Xiao

**Affiliations:** Department of Nephrology, Metabolism and Immunology Laboratory for Urological Diseases, The First Affiliated Hospital of Chongqing Medical University, Chongqing, China; Department of Cardiology, The First Affiliated Hospital of Chongqing Medical University, Chongqing, China; Department of Cardiology, The Second Affiliated Hospital of Chongqing Medical University, Chongqing, China; Department of Critical Care Medicine, The Second Affiliated Hospital of Chongqing Medical University, Chongqing, China; Department of Cardiology, The First Affiliated Hospital of Chongqing Medical University, Chongqing, China; Department of Nephrology, Metabolism and Immunology Laboratory for Urological Diseases, The First Affiliated Hospital of Chongqing Medical University, Chongqing, China; Department of Cardiology, Chongqing University Central Hospital, Chongqing Emergency Medical Center, Chongqing, China; Department of Cardiology, Chongqing University Central Hospital, Chongqing Emergency Medical Center, Chongqing, China

**Keywords:** adverse outcomes, chronic kidney disease, clonal hematopoiesis, coronary artery lesions

## Abstract

**Background:**

Patients with chronic kidney disease (CKD) exhibit an extremely high prevalence of coronary artery disease. Clonal hematopoiesis of indeterminate potential (CHIP) and CKD share pathological features such as aging, chronic inflammation, and accelerated atherosclerosis. Their coexistence can synergistically exacerbate vascular damage and increase coronary risk. However, the association between CHIP and specific coronary lesions in CKD populations has not been reported, and its relationship with cardiovascular events remains controversial.

**Methods:**

A total of 151 patients with CKD who underwent coronary angiography were prospectively included. To evaluate the status of CHIP, we utilized high-depth targeted sequencing, and measured serum inflammatory factor levels. Furthermore, we systematically followed up these patients to document the occurrence of adverse clinical events.

**Results:**

CHIP was identified in 65 (43.0%) CKD patients, with the carrier rate steadily rising with age. The CHIP subjects had higher rates of left circumflex stenosis, three-vessel disease, and Gensini scores than non-CHIP patients (all *P* < .05). After adjusting for relevant clinical risk factors, the presence of CHIP continued to show an independent association with three-vessel disease [odds ratio 2.26, 95% confidence interval (CI) 1.07–4.75; *P* = .032]. The survival analysis indicated that CHIP, along with non-*DNMT3A* mutations and a larger clone size (variant allele frequency ≥0.10), correlated with the primary composite endpoint (*P* < .01). Even after controlling for various clinical variables, the CHIP status still demonstrated an independent association with the primary composite endpoint (hazard ratio 2.02, 95% CI 1.11–3.67; *P* = .022).

**Conclusions:**

CHIP was associated with the severity of coronary lesions and unfavorable clinical outcomes in patients with CKD.

## Graphical Abstract

**Graphical Abstract:**
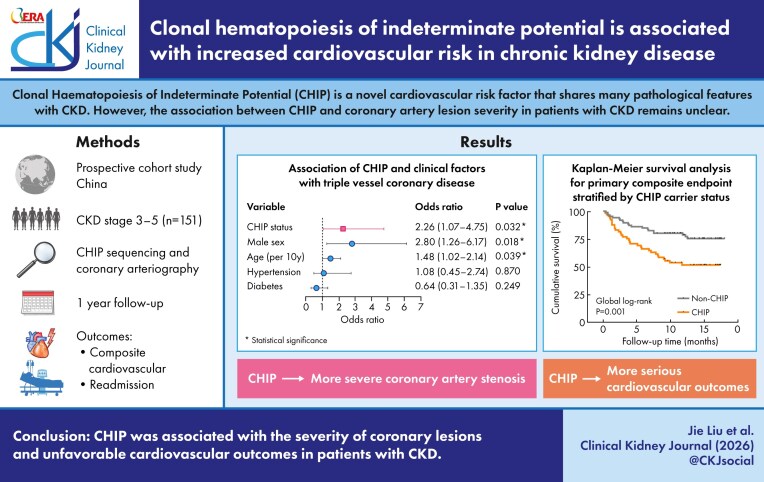

KEY LEARNING POINTS **What was known:**

- Cardiovascular disease is considered as one of the leading causes of death in chronic kidney disease (CKD).
- Clonal hematopoiesis of indeterminate potential (CHIP) is a new risk factor for cardiovascular disease.
- Prior studies involving CKD populations have reported inconsistent associations between CHIP and cardiovascular events.

**This study adds:**

- This is the first study to report the association between CHIP and the severity of coronary artery disease assessed by coronary angiography in CKD patients.

**Potential impact:**

- CHIP may have prognostic value for cardiovascular events in CKD patients. In certain high-risk CKD populations, CHIP assessment may complement existing cardiovascular risk evaluation strategies.

## INTRODUCTION

Chronic kidney disease (CKD) is a prevalent condition often found alongside cardiovascular diseases, such as coronary artery disease, stroke, heart failure, and peripheral vascular disease [1–4]. Cardiovascular disease stands out as the leading cause of mortality in CKD, and the likelihood of developing this condition rises significantly as kidney disease progresses [1, 5].

Clonal hematopoiesis of indeterminate potential (CHIP) represents a somatic pathogenic occurrence. This phenomenon arises when hematopoietic stem cells undergo mutations that grant them a competitive advantage, leading to the clonal proliferation of circulating blood cells. Such mutations play a significant role in the development of myelodysplastic syndromes (MDSs) and acute myeloid leukemia [6, 7]. Among the various mutations, the driver genes *DNMT3A* and *TET2* are the most commonly altered, accounting for ∼70% of the identified somatic mutation sites [8]. Research involving animal models and clinical investigations indicates that CHIP may be implicated in the onset and progression of conditions such as atherosclerosis, heart failure, and hypertension (HTN) [9–14].

CHIP-associated somatic mutations mechanistically modify the transcriptional profile of myeloid cells, pushing them into a hyperinflammatory state. These mutant immune cells penetrate the vascular walls and excessively release pro-inflammatory cytokines, which worsen endothelial dysfunction and directly speed up the advancement of atherosclerotic plaques. However, the association between these factors is uncertain in CKD patients. While one study found no association between CHIP and coronary artery calcification or major adverse cardiovascular events [15], another research indicated an increased risk of adverse outcomes associated with CHIP [16]. Notably, both CKD and CHIP share some pathophysiological process like older age, chronic inflammation, and accelerated atherosclerosis. The convergence of these two conditions may potentially potentiate their individual impact on vascular damage, leading to a synergistic increase in coronary risk. Despite this biological plausibility, it is not yet clear if CHIP is connected to the severity of coronary lesions in CKD.

Therefore, this study aimed to examine any association between CHIP and severity of coronary artery stenosis as measured by coronary angiogram, and CHIP and its subtypes with cardiovascular event risk.

## MATERIALS AND METHODS

### Study population

**Figure 1:**
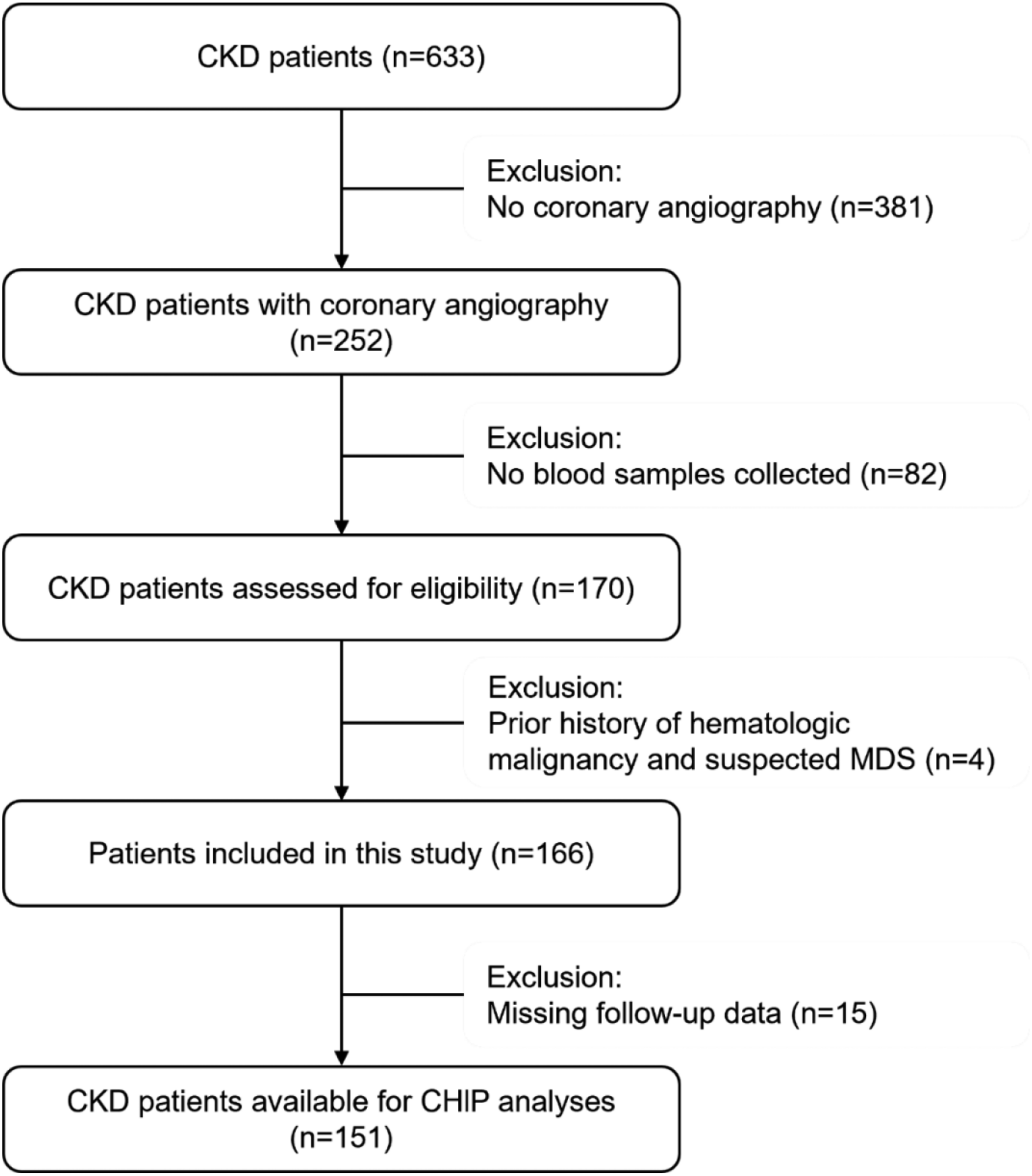
Flowchart of study population. Total numbers and reasons for exclusion are detailed at each step.

Between December 2023 and October 2024, we screened 633 patients with CKD stages 3–5 who underwent clinically indicated invasive coronary angiography across two centers (the First Affiliated Hospital of Chongqing Medical University and Chongqing Emergency Medical Center). Patients lacking blood samples or follow-up data were excluded. To minimize confounding and strictly differentiate CHIP from overt hematologic malignancies, patients with a documented history of leukemia or MDSs were excluded based on electronic medical records. Furthermore, suspected MDS was clinically ruled out by evaluating routine hemogram parameters (white blood cell count, hemoglobin, platelets, and mean corpuscular volume); essentially, patients exhibiting unexplained macrocytosis or severe multilineage cytopenias were excluded. After applying these criteria, the final cohort for CHIP analysis comprised 151 patients (Fig. 1 ). A formula modified specifically for the Chinese population was used for estimated glomerular filtration rate (eGFR): eGFR = 175 × (serum creatinine)^−1.234^ × (age)^−0.179^ × 0.79 (if female) [17]. The stages of CKD were categorized based on GFR ranges as outlined in guidelines (measured in ml/min/1.73 m^2^): stage 1 (≥90), stage 2 (60–89), stage 3 (30–59), stage 4 (15–29), and stage 5 (< 15 or requiring dialysis) [18]. Initially, patients received an ethylenediaminetetraacetic acid (EDTA) anticoagulant tube from which blood samples were collected peripherally. The Institutional Review Boards of the two institutions approved the protocol of the study.

**Figure 2:**
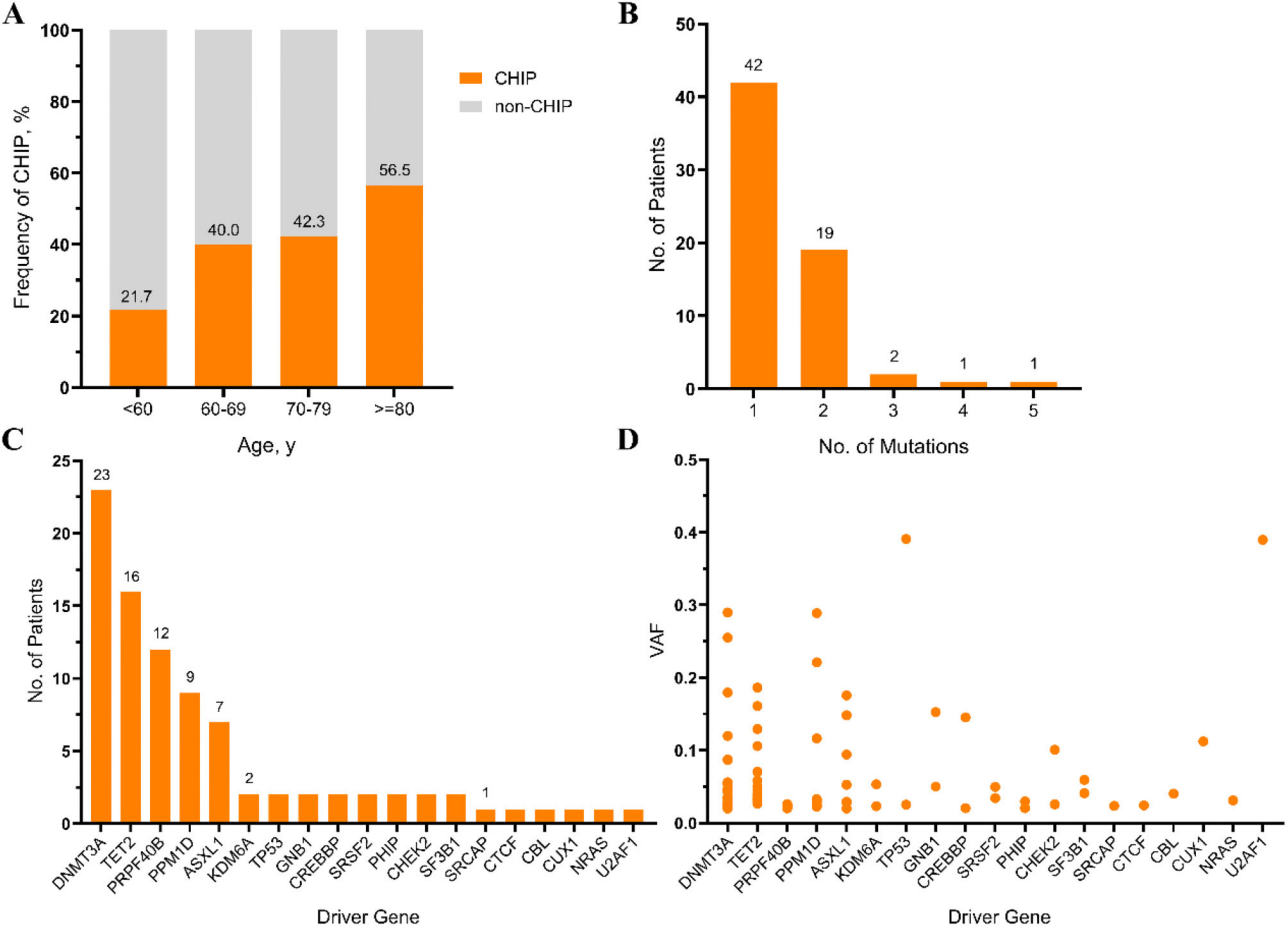
Prevalence and spectrum of CHIP-related driver mutations and genes in patients with CKD. **(A)** Age–related distribution frequency of CHIP. **(B)** Total number of patients with different numbers of mutations. **(C)** Number of patients carrying driver genes. **(D)** VAF distribution across driver genes.

### Collection of clinical data and biochemical parameter assessment

The hospital’s electronic medical records system provided information on demographic characteristics and comorbidities. Blood samples were taken for fasting of each subject and subsequently analysed on standard automated analyzers. Tests performed in the laboratory included blood factors, lipids, renal indicators, bone metabolism, as well as inflammatory and cardiac markers. All assays were performed by laboratory personnel who were blinded to the clinical outcomes. Baseline medication use was documented at enrollment. Suspected etiologies of CKD were categorized into four mutually exclusive groups based on clinical comorbidities: (i) both diabetes mellitus (DM) and HTN; (ii) DM only; (iii) HTN only; and (iv) other/unknown (patients without DM or HTN, potentially representing cases of primary glomerulonephritis or other etiologies without histopathological confirmation).

### Coronary angiography

Coronary angiography was performed according to standard clinical protocols and visually interpreted by experienced interventional cardiologists, with additional independent blinded review by senior physicians. All readers were blinded to the patients’ CHIP status. Stenosis severity was quantified as the percentage of luminal narrowing relative to the adjacent normal reference vessel diameter. The Gensini score was calculated to assess the overall burden of coronary atherosclerosis. Obstructive coronary artery disease is characterized by a stenosis of 50% or more. Patients were categorized into single-vessel, double-vessel, or triple-vessel disease based on the involvement of the major epicardial arteries: the left anterior descending artery (LAD), left circumflex artery (LCx), and right coronary artery (RCA) [19]. To evaluate the overall extent of coronary atherosclerosis, the Gensini score was utilized. The total score represents the sum of individual lesion scores. Specifically, each lesion score was derived by multiplying the severity score (determined by the degree of stenosis) by a weighting factor corresponding to the functional significance of the lesion’s location [20–22].

### CHIP identification

We identified somatic mutations associated with CHIP by targeted deep sequencing of 24 candidate genes. Genomic DNA from peripheral blood was sequenced to a mean depth of ∼2680×, providing sufficient sensitivity for variant detection. The human reference genome (hg38) was used to align reads, and ANNOVAR was employed for variant annotation. Individuals identified with somatic mutations and a variant allele fraction (VAF) of 2% or higher were categorized as CHIP carriers [12, 23, 24]. We applied rigorous filtering criteria to exclude nonsomatic variants: single-nucleotide polymorphisms with a minor allele frequency ≥1% in population databases (1000 Genomes, ExAC, or gnomAD); synonymous, nonframeshift indels, and noncoding variants to ensure a focus on high-confidence protein-altering alterations within exons; technical artifacts appearing in >8% of the cohort and potential germline variants with VAFs of 40%–60% or ≥90% [12, 23, 25]. Further details are available in the Supplementary methods section in the Supplementary material.

### Quantification of inflammatory cytokines by enzyme-linked immunosorbent assay

Plasma levels of interleukin-1β (IL-1β) (Proteintech, Wuhan, China), interleukin-6 (IL-6), and monocyte chemoattractant protein-1 (MCP-1) (Boster, Wuhan, China) were measured using enzyme-linked immunosorbent assay kits according to the manufacturers’ instructions. Inflammatory cytokine measurements were available in a subset of patients due to sample availability and quality control constraints, including hemolysis and insufficient blood volume for complete testing.

### Outcome evaluation

Clinical outcomes were ascertained through scheduled follow-up every 3 months (via outpatient visits or telephone interviews). The main endpoint comprised a combination of cardiovascular mortality, nonfatal myocardial infarction, nonfatal stroke, and admissions due to heart failure. Heart failure readmission was characterized as a hospitalization required due to new or exacerbated signs and symptoms of heart failure, substantiated by noninvasive imaging findings or elevated levels of N-terminal pro-B-type natriuretic peptide (NT-proBNP), along with a discharge diagnosis confirming congestive heart failure [26].

### Statistical analysis

Statistical analyses were conducted utilizing SPSS version 26.0 (IBM Corp, Armonk, NY). Continuous variables were represented as mean ± standard deviation (SD) or median [interquartile range (IQR)], depending on the normality assessed by the Shapiro–Wilk test, and were compared using either Student’s *t*-test or the Mann–Whitney *U* test. Categorical variables were articulated as frequencies (%) and analysed using the chi-square test. Survival curves were produced following the Kaplan–Meier methodology and assessed through the log-rank test. To identify the independent relationship between CHIP and outcomes, multivariate Cox proportional hazards models were employed, reporting hazard ratios (HRs) alongside 95% confidence intervals (CIs). A two-sided *P* < .05 was considered significant.

## RESULTS

### Baseline characteristics and CHIP mutation profile

From December 2023 to October 2024, a total of 151 CKD patients undergoing coronary angiography were enrolled, with the majority presenting at CKD stage 3 (72.8%). Targeted sequencing identified the occurrence of CHIP in 65 individuals (43.0%), a prevalence significantly higher than that observed in previous cohorts (CRIC: 28.0%; BioVU: 19.0%). This discrepancy likely reflects the characteristics of this older, uniformly cardiovascular-diseased population (Supplementary Table S2). The prevalence of CHIP demonstrated a positive correlation with age, increasing from 21.7% in patients under 60 years to 56.5% in those aged 80 years and older (Fig. 2A). Among the patients affected (*n* = 42), the majority carried a single driver mutation (Fig. 2B). The genes with the highest mutation frequencies included *DNMT3A* (*n* = 23) and *TET2* (*n* = 16), with *PRPF40B, PPM1D*, and *ASXL1* following (Fig. 2C and D). Moreover, the baseline etiologies of CKD, like DM and HTN, did not reveal any significant differences between the CHIP and non-CHIP groups (*P* > .05; Supplementary Table S3).

### Clinical characteristics of CHIP carriers

Patients diagnosed with CHIP exhibited a notably greater age than those not affected by CHIP [median 78.0 (69.0–82.0) vs 72.0 (60.8–78.3) years; *P* = .001]. Furthermore, those with CHIP showed reduced levels of triglycerides [1.2 (0.9–1.8) vs 1.6 (1.0–2.3) mmol/l; *P* = .034]. No other significant variances in clinical features were observed between the two groups (Table 1).

**Table 1:** Baseline characteristics of the study cohort.

Characteristic	Total	non-CHIP (*n* = 86)	CHIP (*n* = 65)	*P*-value
**Demographic and clinical characteristics**				
Age, years	73.0 (67.0–81.0)	72.0 (60.8–78.3)	78.0 (69.0–82.0)	.001
Male, *n* (%)	103 (68.2%)	56 (65.1%)	47 (72.3%)	.347
CKD stage				.517
3	110 (72.8%)	60 (69.8%)	50 (76.9%)	
4	22 (14.6%)	13 (15.1%)	9 (13.8%)	
5	19 (12.6%)	13 (15.1%)	6 (9.2%)	
HTN, *n* (%)	121 (80.1%)	71 (82.6%)	50 (76.9%)	.390
DM, *n* (%)	79 (52.3%)	44 (51.2%)	35 (53.8%)	.744
Smoking, *n* (%)	74 (49.0%)	43 (50.0%)	31 (47.7%)	.740
**Hematologic parameters**				
WBC, ×10^9^/l	7.4 (5.7–9.4)	7.5 (5.7–9.4)	7.1 (5.5–9.5)	.823
RBC, ×10^12^/l	4.0 ± 0.8	4.1 ± 0.7	3.9 ± 0.9	.219
Hemoglobin, g/l	117.3 ± 23.0	120.2 ± 21.3	113.3 ± 24.8	.068
Platelets, ×10^9^/l	179.0 (136.5–211.0)	178.0 (135.5–204.5)	184.0 (133.5∼252.0)	.708
MCV, fl	93.0 (88.2–96.1)	92.6 (88.0–95.6)	93.5 (88.5–96.2)	.549
**Metabolic and lipid profile**				
TC, mmol/l	3.7 (3.0–4.5)	3.8 (3.1–4.9)	3.6 (2.8–4.3)	.350
HDL-C, mmol/l	1.1 ± 0.3	1.1 ± 0.3	1.1 ± 0.3	.432
LDL-C, mmol/l	2.1 (1.5–2.7)	2.1 (1.6–2.5)	2.0 (1.3–2.8)	.889
TG, mmol/l	1.4 (0.9–2.1)	1.6 (1.0–2.3)	1.2 (0.9–1.8)	.034
**Renal function and electrolytes**				
BUN, mmol/l	10.7 (7.8–14.5)	9.5 (7.4–14.7)	11.4 (8.3–14.9)	.132
Creatinine, µmol/l	134.5 (120.0–186.3)	136.0 (117.8–192.5)	133.5 (123.8–181.3)	.969
Uric acid, mg/dl	415.5 (322.0–500.8)	417.5 (318.5–518.0)	413.5 (329.5–475.0)	.722
eGFR, ml/min/1.732 m^2^	45.9 (28.5–54.2)	46.6 (27.4–56.0)	45.0 (32.0–52.4)	.559
Proteinuria, *n* (%)	68 (45.0%)	41 (47.7%)	27 (41.5%)	.453
Ca^2+^, mmol/l	2.2 (2.1–2.3)	2.2 (2.1–2.3)	2.2 (2.1–2.3)	.546
Alkaline phosphatase, U/l	76.5 (64.8–103)	76.5 (64–107.3)	76.5 (66–98.3)	.814
**Cardiac biomarkers**				
NT-proBNP, pg/ml	2110.0 (390.0–6776.0)	1827.1 (242.0–5796.3)	2805.5 (688.3–9347.5)	.156
**Medical therapy**				
ACEI/ARB/ARNI, *n* (%)	85 (56.3%)	48 (55.8%)	37 (56.9%)	.892
Statins, *n* (%)	134 (88.7%)	74 (86.0%)	60 (92.3%)	.228
**Echocardiography parameters**				
LVEDD	50.0 (45.3–55.8)	48.0 (45.0–53.0)	50.0 (45.0–58.0)	.164
LVESD	34.0 (30.0–42.8)	33.0 (30.0–43.5)	35.0 (29.5–41.0)	.792
IVSd	11.0 (10.0–12.0)	10.0 (9.8–11.9)	11.0 (10.0–12.0)	.451
LVPWd	10.0 (10.0–11.0)	10.0 (9.0–11.0)	10.0 (10.0–11.0)	.178
LVEF, %	58.0 (47.0–62.3)	59.0 (47.0–63.5)	58.0 (46.5–60.0)	.072

Data are presented as mean ± SD, median (IQR), or count (%).

ACEI, angiotensin-converting enzyme inhibitor; ARB, angiotensin II receptor blocker; ARNI, angiotensin receptor-neprilysin inhibitor; BUN, blood urea nitrogen; HDL-C, high-density lipoprotein cholesterol; IVSd, interventricular septal thickness at end-diastole; LDL-C, low-density lipoprotein cholesterol; LVEDD, left ventricular end-diastolic diameter; LVESD, left ventricular end-systolic diameter; LVEF, left ventricular ejection fraction; LVPWd, left ventricular posterior wall thickness at end-diastole; MCV, mean corpuscular volume; RBC, red blood cell count; TC, total cholesterol; TG, triglyceride; WBC, white blood cell count.

### CHIP and coronary artery lesions

**Figure 3:**
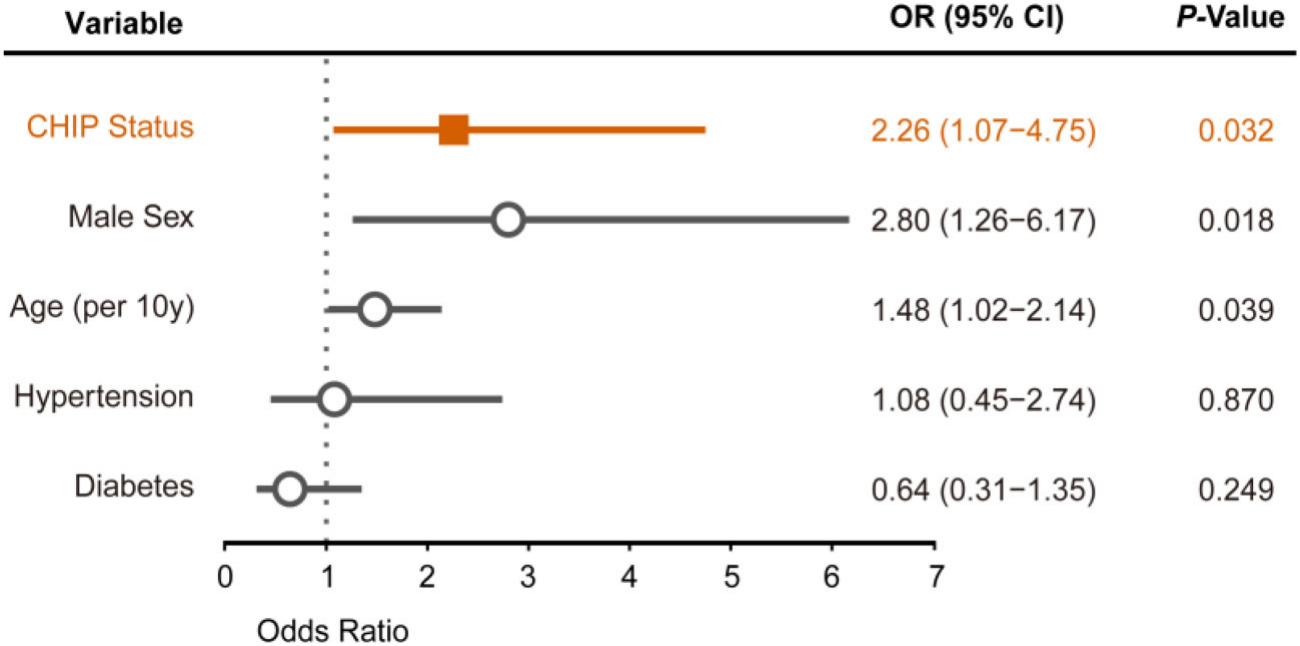
Association between CHIP status and the risk of triple-vessel coronary disease. Forest plot of multivariable logistic regression analysis evaluating the association of CHIP status and clinical risk factors with triple-vessel coronary disease. Markers represent point estimates of ORs, and error bars indicate 95% CIs.

To understand how CHIP relates to coronary atherosclerosis, we looked at data on stenotic lesions seen in coronary angiography. The distribution of the total number of coronary stenoses was shifted to the right in CHIP patients compared to non-CHIP patients [2.0 (1.0–3.0) vs 2.0 (1.0–2.3); *P* = .035]. CHIP subjects exhibited higher rates of LCx stenosis (60.0% vs 40.7%; *P* = .019), triple-vessel disease (47.7% vs 24.4%; *P* = .003), and Gensini scores [60.5 (31.3–108.5) vs 38.0 (24.0–63.5); *P* = .011] compared to non-CHIP patients (Table 2). After adjusting for clinical risk factors such as age, sex, diabetes, and HTN, the presence of three-vessel disease remained independently linked to CHIP [odds ratio (OR) 2.26, 95% CI 1.07–4.75; *P* = .032] (Fig. 3).

**Table 2:** Associations between CHIP and coronary artery lesion characteristics.

Characteristic	Total	non-CHIP (*n* = 86)	CHIP (*n* = 65)	*P*-value
Total number of significant stenotic segments, *n*	2.0 (1.0, 3.0)	2.0 (1.0, 2.3)	2.0 (1.0, 3.0)	.014
LM stenosis, *n* (%)	14 (9.3%)	8 (9.3%)	6 (9.2%)	.988
LAD stenosis, *n* (%)	116 (76.8%)	62 (72.1%)	54 (83.1%)	.113
LAD TIMI flow grade	3.0 (3.0, 3.0)	3.0 (3.0, 3.0)	1.5 (3.0, 3.0)	.134
LCx stenosis, *n* (%)	74 (49.0%)	35 (40.7%)	39 (60.0%)	.019
LCx TIMI flow grade	3.0 (3.0, 3.0)	3.0 (3.0, 3.0)	3.0 (3.0, 3.0)	.039
RCA stenosis, *n* (%)	96 (63.6%)	52 (60.5%)	44 (67.7%)	.361
RCA TIMI flow grade	3.0 (3.0, 3.0)	3.0 (3.0, 3.0)	3.0 (3.0, 3.0)	.098
Single-vessel disease, *n* (%)	34 (22.5%)	22 (25.6%)	12 (18.5%)	.300
Double-vessel disease, *n* (%)	48 (31.8%)	32 (37.2%)	16 (24.6%)	.100
Triple-vessel disease, *n* (%)	52 (34.4%)	21 (24.4%)	31 (47.7%)	.003
Gensini score	44.5 (26.1−89.8)	38.0 (24.0−63.5)	60.5 (31.3−108.5)	.011
Coronary stent placement, *n* (%)	79 (52.3%)	45 (52.9%)	34 (52.3%)	.939

LM, Left Main coronary artery.

### CHIP and clinical events

**Figure 4:**
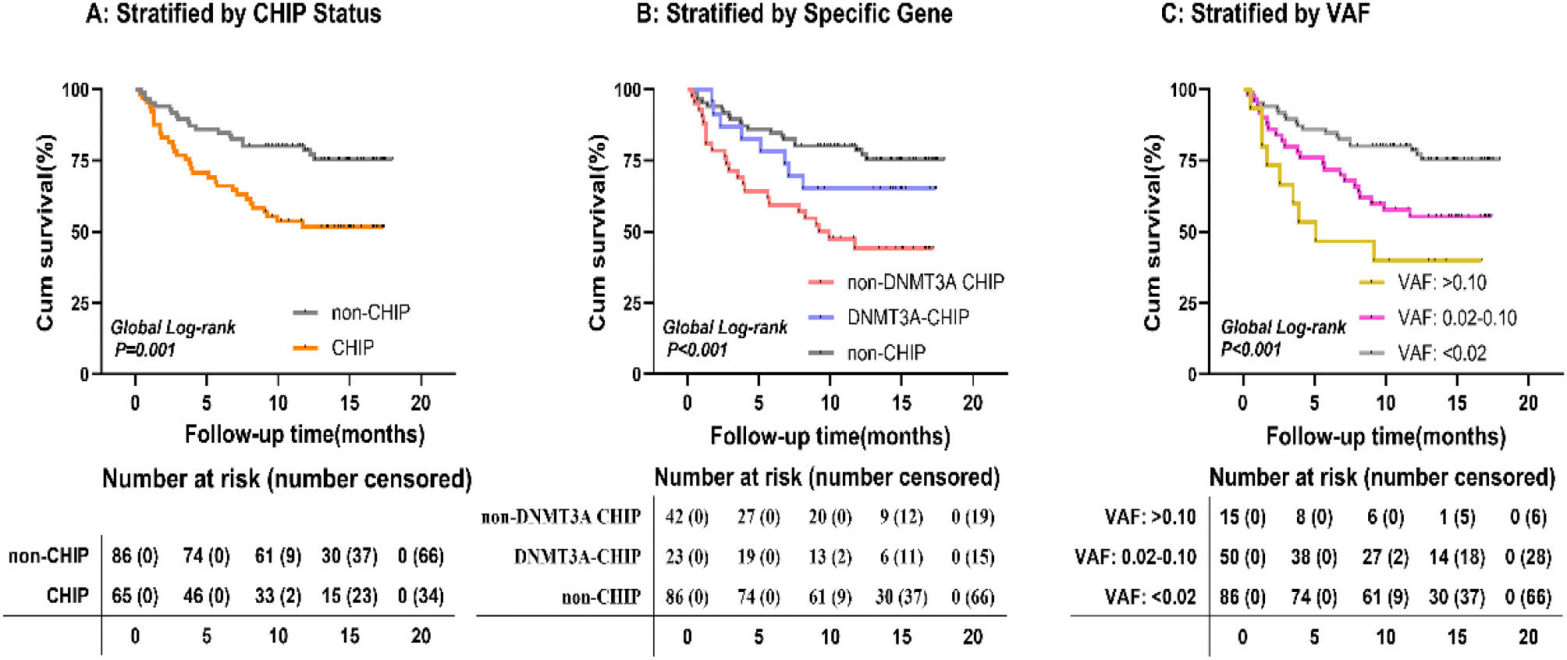
Kaplan–Meier survival analysis for the primary composite endpoint stratified by CHIP characteristics. Kaplan–Meier survival curves for freedom from the composite endpoint according to **(A)** overall CHIP carrier status; **(B)** specific driver gene mutations (*DNMT3A* vs non-*DNMT3A* vs non-CHIP carriers); and **(C)** VAF tiers (VAF ≥ 0.10 vs VAF 0.02–0.10 vs VAF < 0.02). Differences between groups were assessed by the log-rank test.

After a median follow-up period of 12 months, 51 patients experienced the composite outcome. The Kaplan–Meier analysis indicated that individuals carrying CHIP had considerably lower survival rates compared to noncarriers (log-rank *P* = .001) (Fig. 4A). Importantly, the most severe outcomes were identified in patients without *DNMT3A* mutations (Fig. 4B) or in those exhibiting larger clone sizes (VAF ≥ 0.10) (Fig. 4C), both categories demonstrating log-rank *P*-values below .001. The multivariable Cox regression analysis revealed that CHIP was independently linked to an elevated risk of the composite cardiovascular event, even after controlling for possible confounding factors (HR 2.02, 95% CI 1.11–3.67; *P* = .022) (Supplementary Table S5).

### Inflammatory profile of CHIP carriers

We compared patients with and without CHIP for circulating inflammatory markers to explore potential mechanisms. CHIP carriers had higher level of IL-1β, IL-6, and high-sensitivity C-reactive protein (hs-CRP) compared to other patients who did not carry these mutations (*P* < .05, Supplementary Figure S1). To further evaluate whether the elevated cytokine levels were primarily driven by the decline in renal function, we conducted an exploratory correlation analysis between eGFR and cytokines. Our analysis revealed no significant correlations between eGFR and the levels of IL-1β, IL-6, hs-CRP, or MCP-1 (Supplementary Table S7). Additionally, in a subgroup analysis, we found that hs-CRP significantly inversely correlated with triglycerides (*r* = −0.465, *P* = .001) and total cholesterol (*r* = −0.291, *P* = .039) (Supplementary Table S8).

## DISCUSSION

This prospective cohort study investigated the link between CHIP, the extent of coronary artery lesions, and cardiovascular outcomes among CKD patients undergoing coronary angiography. While the association between CHIP and cardiovascular events is well known, our research offers new insights into anatomical features, demonstrating that individuals with CHIP exhibited more severe coronary artery disease. This was reflected in a higher occurrence of three-vessel disease and LCx artery stenosis when compared to those without CHIP. Additionally, our analyses suggested that mutations beyond *DNMT3A*, along with an elevated VAF, correlated with a greater incidence of cardiovascular events. These results indicate that CHIP could potentially serve as a marker for the complexity of coronary disease and adverse outcomes within the CKD patient population.

CHIP was significantly associated with increased severity of coronary artery stenosis in the CKD population, which aligns with previous findings in other high-risk groups such as patients undergoing cardiac catheterization and individuals living with HIV [27, 28]. To intuitively reflect the overall burden of coronary disease, we take triple-vessel disease as the core indicator for clinical stratification. We observed that CHIP carriers had significantly increased proportions of triple-vessel disease and stenosis in the LCx artery compared with noncarriers. The anatomical preference for LCx likely results from a synergistic action of systemic inflammation by CHIP and endothelial shear stress of LCx, contributing to rapid regional plaque progression [29]. In addition, significant LCx involvement indicates a trajectory toward diffuse multivessel disease, consistent with the rapid atherosclerotic phenotype of CHIP. Although CHIP has been recognized to be linked to multisystem adverse outcomes in CKD patients [16, 30, 31], there remains limited clinical evidence on its association with anatomical coronary lesions. Through coronary angiography, this study is the pioneer to demonstrate the anatomical association of CHIP with complex coronary lesions in CKD. Thus, it is the first clinical evidence that sheds light on this potential pathophysiological mechanism.

CHIP is associated with an elevated risk of adverse clinical events in patients with CKD. The risk significantly appears to be further augmented by presence of mutations other than *DNMT3A*, and higher VAF. This highlights significant genetic heterogeneity and a dose-response effect [10, 12, 16, 23, 24, 32]. The findings suggest that the driver gene and the extent of the clonal expansion determine the ultimate CHIP effect on cardiovascular risk. This shows the clinical utility of identifying CHIP early for accurate risk assessment and intervention.

This elevated risk is likely rooted in a synergistic “double-hit” interaction between CKD and CHIP. Alterations in epigenetic regulators like *TET2* and *DNMT3A* drive the hypersensitization of the NLRP3 inflammasome in macrophages [33, 34]. At the same time, the uremic setting associated with CKD imparts a growth advantage to these mutated clones, while also having a potent inflammatory capacity. In this uremic environment, macrophages with mutations release cytokines like IL-1β and IL-6 quickly and for a long time [34–36]. This localized and systemic inflammatory response dramatically accelerates lipid accumulation, induces profound endothelial dysfunction, and promotes significant plaque instability. Ultimately, this immune-mediated plaque vulnerability, aggravated by the cardio-renal pathogenic microenvironment, solidly explains the severe coronary lesions and frequent acute cardiovascular events occurring in CHIP carriers.

At a median follow-up duration of 12 months, the cumulative rate of composite events and the prevalence of CHIP were found to be markedly higher in our study group compared to what is typically reported in most nondialysis CKD registries [16]. This notable difference is primarily attributed to the unique high-risk clinical characteristics and specific disease stages present in our participant population. The patients included in our cohort, who were referred for coronary angiography, were predominantly older, exhibited advanced coronary anatomical complexity, and faced a significant burden of CKD and cardiovascular disease (CVD). Furthermore, the majority of these participants (72.8%) were diagnosed with stage 3 CKD. In contrast to end-stage renal disease, which is largely influenced by medial calcification, the progression of moderate CKD is primarily driven by inflammation-related atherosclerosis [37, 38]. The unique cardiovascular profile during the acute phase, when combined with severe nontraditional cardio-renal risk factors such as the uremic environment, chronic inflammation, and oxidative stress, produces a significantly pathological microenvironment. Such conditions markedly favor the selective expansion of mutant clones, which helps to elucidate the unexpectedly high prevalence of CHIP. Consequently, the current study effectively illustrates the interplay between traditional cardiovascular risk factors and this specifically tailored cardio-renal pathogenic microenvironment, which is further influenced by elevated event rates and CHIP.

We observed lower triglyceride levels in CHIP carriers despite their increased inflammatory state. This pattern may be explained by the inflammation-driven “lipid paradox” characteristic of CKD [39]. This is further evidenced by markedly raised concentrations of IL-1β, IL-6, and hs-CRP. Also, hs-CRP correlated inversely with triglycerides and total cholesterol. These results imply that CHIP may exert an added inflammatory burden aside from the systemic inflammation already present in CKD. While these cytokines are likely to drive atherosclerotic progression, they may also partially reflect the extent of existing coronary lesions. In general, CHIP may have a dual pathogenic role in accelerating coronary atherosclerosis and CKD by inducing both local inflammation and fibrotic remodeling.

We acknowledge several important limitations. First, the small sample size and highly selected cohort of symptomatic CKD patients undergoing coronary angiography limit statistical power and generalizability, and the lack of an external validation cohort warrants cautious interpretation. Second, the observational design and relatively short median follow-up of 12 months preclude causal inference and limit assessment of long-term prognostic significance. Third, residual confounding cannot be excluded. In particular, the absence of biopsy-confirmed CKD etiologies, an age-matched control group with preserved renal function (eGFR > 60), and screening for concurrent clonal or autoimmune disorders limits our ability to isolate the independent effects of CHIP. Finally, inflammatory biomarkers were examined solely in a limited subgroup, and the absence of corrections for multiple comparisons heightens the chance of false-positive results. Consequently, these findings ought to be viewed as descriptive and as a basis for forming hypotheses, necessitating validation through extensive, multicenter prospective studies.

In conclusion, CHIP is significantly associated with an aggravated coronary artery disease burden and elevated cardiovascular risk in patients with CKD. Notably, non-*DNMT3A* mutations and higher VAF confer particularly high risk, highlighting the genetic heterogeneity of cardiovascular outcomes. While CHIP status shows promise as a complementary risk stratification tool, further prospective validation is required to establish its clinical utility.

## Supplementary Material

sfag141_Supplemental_File

## Data Availability

The datasets used and/or analysed during the current study are available from the corresponding author on reasonable request.
