# Supplementary material for: Clonal hematopoiesis of indeterminate potential is associated with increased cardiovascular risk in chronic kidney disease
Source: Clin Kidney J. 2026 May 8;19(6):sfag141. doi: 10.1093/ckj/sfag141 (PMC13223406; doi:10.1093/ckj/sfag141)
Supplement: sfag141_Supplemental_File [file sfag141_supplemental_file.docx]

**Supplementary Methods: CHIP Identification**

**Library Preparation and Sequencing**

Genomic DNA was extracted from peripheral blood leukocytes. Targeted sequencing libraries were constructed using the TargetSeq® hybridization capture system (iGeneTech, China) targeting the complete coding regions of 24 myeloid driver genes (Supplementary Table S1). Sequencing was performed on the DNBSEQ-T7 platform with 150-bp paired-end reads. The average sequencing depth across target regions was 2,679.5× (median 2,509×), with 99.8% of target bases covered >500×.

**Variant Calling and Filtering**

CHIP was defined as the presence of somatic mutations with a variant allele fraction (VAF) ≥2%. The limit of detection (LOD) was set at 1%. Sequence reads were aligned to the hg38 reference genome using the Genome Analysis Toolkit (GATK). Variant calling was performed using GATK Mutect2, and variants were annotated with ANNOVAR. We adopted a stringent filtering pipeline to distinguish somatic mutations from germline polymorphisms and sequencing artifacts, referencing established protocols[1-3].

**Quality Control**

To enrich for true somatic events, we applied a series of stringent filtering criteria to remove likely non-somatic variants. First, common single-nucleotide polymorphisms were excluded if they had a minor allele frequency of ≥1% in public population databases, including the 1000 Genomes Project, ExAC, and gnomAD. Second, we removed variants unlikely to have substantial functional consequences, including synonymous variants, non-frameshift insertions/deletions, and non-coding variants, thereby restricting the analysis to high-confidence protein-altering alterations located within exonic regions. Finally, we excluded recurrent technical artifacts detected in >8% of the cohort, as well as variants considered likely to be of germline origin based on their variant allele frequencies, specifically those with VAFs of 40–60% or ≥90%.

Supplementary Figure S1. Levels of inflammatory biomarkers stratified by CHIP status.


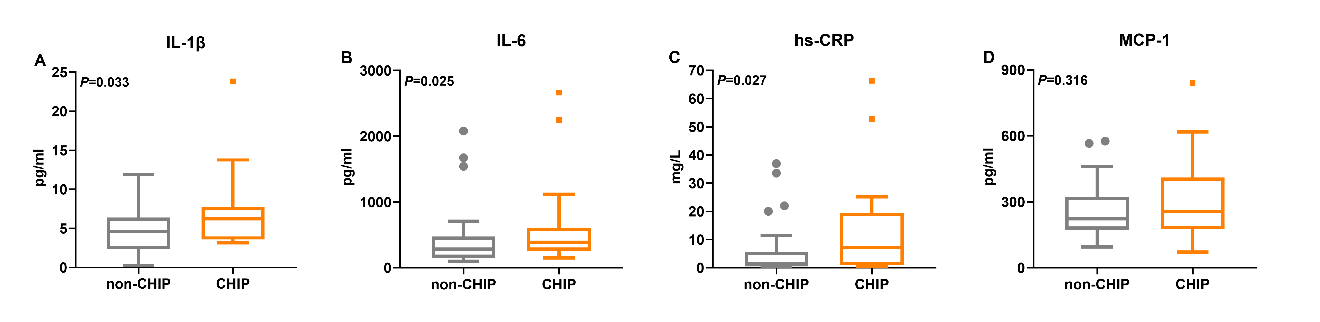


Plasma levels of (A) interleukin-1β (IL-1β, n = 56), (B) interleukin-6 (IL-6, n = 63), (C) high-sensitivity C-reactive protein (hs-CRP, n = 52), and (D) monocyte chemoattractant protein-1 (MCP-1, n = 70) in participants without and with CHIP. Boxes show the IQR, horizontal lines indicate the median, whiskers extend to 1.5 × IQR, and dots represent outliers. P-values were calculated using the Mann-Whitney U test.

Supplementary Table S1. List of Genes Detected by Targeted Deep Sequencing

| ASXL1 | DNMT3A | PHIP | STAG1 |
| --- | --- | --- | --- |
| BCOR | GNB1 | PPM1D | SUZ12 |
| CBL | JAK2 | PRPF40B | TP53 |
| CHEK2 | KDM6A | TET2 | CUX1 |
| CREBBP | KIT | SF3B1 | NRAS |
| CTCF | MPL | SRCAP | SRSF2 |

Supplementary Table S2. Comparison of CHIP prevalence and baseline characteristics across CKD cohorts.

| Characteristics | Current Study | CRIC[4] | CanPREDDICT[4] | AASK[4] | BioVU[4] |
| --- | --- | --- | --- | --- | --- |
| Population | CKD (Undergoing CAG) | CKD | CKD | CKD (Hypertensive) | CKD (Biobank) |
| Sample Size (n) | 151 | 1,568 | 1,432 | 209 | 2,445 |
| CHIP (%) | 43.0% | 28.0% | 26.0% | 27.0% | 19.0% |
| VAF | ≥ 2% | ≥ 2% | ≥ 2% | ≥ 2% | ≥ 2% |
| Age (years) | 73.0 (67.0-81.0) | 70 ± 4 | 68 ± 13 | 61 ± 6 | 63 ± 14 |
| Hypertension (%) | 80.1% | 92.0% | 98.0% | 100% | 27.0% |
| Diabetes (%) | 52.3% | 54.0% | 47.0% | 0% | 30.0% |
| CVD (%) | 100% | 43.0% | 33.0% | 52.0% | 26.0% |
| eGFR (ml/min/1.73m²) | 45.9 (28.5-54.2) | 46 ± 15 | 29 ± 10 | 48 ± 12 | 48 ± 19 |

Note: CVD: Cardiovascular disease; eGFR: estimated glomerular filtration rate; VAF: Variant allele frequency.

Supplementary Table S3. Etiology of chronic kidney disease.

| Cause of CKD | non-CHIP (n=86) | CHIP (n=65) | P-value |
| --- | --- | --- | --- |
| Both DM and HTN | 26 (30.2%) | 27 (41.5%) | 0.201 |
| DM Only | 18 (20.9%) | 8 (12.3%) | 0.234 |
| HTN Only | 32 (37.2%) | 19 (29.2%) | 0.312 |
| Other/Unknown | 10 (11.6%) | 11 (16.9%) | 0.354 |

Note: Both DM and HTN: Patients with a documented clinical history of both Diabetes Mellitus and Hypertension. DM Only: Patients with a history of Diabetes Mellitus but without Hypertension. HTN Only: Patients with a history of Hypertension but without Diabetes Mellitus. Other/Unknown: Patients with neither Diabetes Mellitus nor Hypertension. This subgroup likely includes primary renal diseases that could not be histologically confirmed in this cardiovascular setting. P-values were calculated using the Chi-square test. DM, diabetes mellitus; HTN, hypertension.

Supplementary Table S4. Comparison of baseline characteristics between patients with and without Triple-Vessel Disease (TVD).

| **Characteristic** | **Non-TVD (n=99)** | **TVD (n=52)** | ***P-*value** |
| --- | --- | --- | --- |
| **Demographic and Clinical Characteristics** |  |  |  |
| Age, years | 72.0 (66.0–80.0) | 76.5 (68.8–82.0) | 0.051 |
| Male, n (%) | 62 (62.6%) | 41 (78.8%) | 0.042 |
| Smoking, n (%) | 51 (51.5%) | 23 (44.2%) | 0.487 |
| Hypertension, n (%) | 80 (80.8%) | 41 (78.8%) | 0.774 |
| Diabetes mellitus, n (%) | 55 (55.6%) | 24 (46.2%) | 0.272 |
| **Metabolic and Lipid Profile** |  |  |  |
| LDL-C, mmol/L | 2.1 (1.5–2.8) | 2.0 (1.4–2.7) | 0.707 |
| **Renal Function** |  |  |  |
| eGFR, mL/min/1.73 m² | 45.9 (29.1–53.9) | 46.9 (28.2–55.0) | 0.742 |
| **Genetic Profile** |  |  |  |
| CHIP carrier, n (%) | 34 (34.3%) | 31 (59.6%) | 0.003 |

Abbreviations: eGFR, estimated glomerular filtration rate; LDL-C, low-density lipoprotein cholesterol.

Supplementary Table S5. Multivariate Cox regression analysis for predictors of the primary composite endpoint.

| **Variable** | **HR** | **95% CI** | ***P-*value** |
| --- | --- | --- | --- |
| CHIP | 2.02 | 1.11–3.67 | 0.022 |
| Age | 1.03 | 1.00–1.06 | 0.058 |
| Sex (Male) | 1.06 | 0.57–1.97 | 0.865 |
| Hypertension | 0.82 | 0.41–1.65 | 0.585 |
| Diabetes | 1.01 | 0.57–1.78 | 0.984 |

The multivariable model was adjusted for age, sex, hypertension, and diabetes simultaneously. Abbreviations: CI, confidence interval; HR, Hazard Ratio.

Supplementary Table S6. Age-stratified and age-restricted analyses

| **Outcome** | **Subgroup** | **HR** | **95% CI** | ***P-*value** |
| --- | --- | --- | --- | --- |
| Composite endpoint | Age <73 | 4.76 | 1.71-13.28 | 0.003 |
|  | Age ≥73 | 1.55 | 0.71-3.39 | 0.271 |
|  | Age <80 | 3.98 | 1.77-8.95 | 0.001 |

Adjusted for sex, hypertension, diabetes mellitus, smoking status, hemoglobin, and eGFR. Age strata were defined by the cohort median age (73 years); age-restricted analyses excluded participants aged ≥80 years.

Supplementary Table S7. Spearman correlation analysis between eGFR and inflammatory cytokines.

| Variable | Spearman's rho (r) | p-value |
| --- | --- | --- |
| IL-1β | -0.068 | 0.618 |
| IL-6 | -0.126 | 0.326 |
| hs-CRP | -0.205 | 0.146 |
| MCP-1 | -0.136 | 0.260 |

Note: eGFR, estimated glomerular filtration rate; IL, interleukin; hs-CRP, high-sensitivity C-reactive protein; MCP-1, monocyte chemoattractant protein-1.

Supplementary Table S8. Spearman correlation analysis between lipid profiles and inflammatory cytokines.

| Variable | hs-CRP | | IL-1β | | IL-6 | | MCP-1 | |
| --- | --- | --- | --- | --- | --- | --- | --- | --- |
|  | r | p | r | p | r | p | r | p |
| TG | -0.465 | 0.001 | -0.071 | 0.621 | -0.198 | 0.163 | -0.111 | 0.438 |
| TC | -0.291 | 0.039 | -0.126 | 0.378 | -0.091 | 0.526 | -0.144 | 0.312 |
| LDL-C | -0.221 | 0.12 | -0.089 | 0.534 | -0.045 | 0.755 | -0.102 | 0.476 |
| HDL-C | -0.134 | 0.348 | -0.012 | 0.934 | -0.112 | 0.434 | -0.056 | 0.696 |

Note: Data represent Spearman's correlation coefficient (r) and the corresponding p-value. TG, triglycerides; TC, total cholesterol; hs-CRP, high-sensitivity C-reactive protein; IL, interleukin; MCP-1, monocyte chemoattractant protein-1; LDL-C, low-density lipoprotein cholesterol; HDL-C, high-density lipoprotein cholesterol.

References

1. Wang S, Hu S, Luo X et al. Prevalence and prognostic significance of DNMT3A- and TET2- clonal haematopoiesis-driver mutations in patients presenting with ST-segment elevation myocardial infarction. EBioMedicine. 2022;78:103964.

2. Dorsheimer L, Assmus B, Rasper T et al. Association of Mutations Contributing to Clonal Hematopoiesis With Prognosis in Chronic Ischemic Heart Failure. JAMA Cardiol. 2019;4(1):25-33.

3. Akhiyat N, Lasho T, Ganji M et al. Clonal Hematopoiesis of Indeterminate Potential Is Associated With Coronary Microvascular Dysfunction In Early Nonobstructive Coronary Artery Disease. Arterioscler Thromb Vasc Biol. 2023;43(5):774-83.

4. Vlasschaert C, Pan Y, Chen J et al. Clonal Hematopoiesis of Indeterminate Potential and Progression of CKD. J Am Soc Nephrol. 2025;36(9):1764-74.
